# The effect of traditional Chinese medicine fumigation and washing as a complementary and alternative therapy on the recovery of joint function after development dysplasia of the hip in children: A protocol for systematic review and meta-analysis

**DOI:** 10.1097/MD.0000000000025686

**Published:** 2021-04-30

**Authors:** Weipeng Zeng, Hanru Mao, Gang Zhou, Kaiqiu Wu, Xinping Liao, Linqian Yun, Jianping Lin

**Affiliations:** a Department of Orthopaedics; b Department of Emergency Surgery, Hainan General Hospital (Hainan Affiliated Hospital of Hainan Medical University), No. 19, Xiuhua Road, Xiuying District, Haikou City 570100, Hainan Province, PR China.

**Keywords:** complementary and alternative therapy, development dysplasia of the hip, meta-analysis, the recovery of joint function, traditional Chinese medicine fumigation and washing

## Abstract

**Background:**

Development dysplasia of the hip (DDH) is a common childhood orthopedic disease in clinic. The cause of DDH is not yet clear. If DDH is not treated promptly or correctly, it will seriously affect the life quality of the child. At present, surgery is the main means of treating older DDH, but it is easy to appear development dysplasia of the hip after surgery, and the joint movement is limited after surgery. For modern medicine, it has not many treatments to solve this problem. As one of the commonly used treatment methods, but the effect of routine functional exercise is not ideal. Traditional Chinese medicine fumigation and washing belongs to the category of Chinese medicine external treatment, which can directly act on the focus. It has the functions of relaxing muscles and tendons and removing obstruction from meridians, activating blood to eliminate stagnation. It has achieved good effects in relieving joint disorders, but it is lack of the high-quality evidence support, so there is controversy about the clinical application of traditional Chinese medicine fumigation and washing. This study will conduct a systematic review to compare the application effect and safety of traditional Chinese medicine fumigation and washing as a complementary and alternative therapy and traditional rehabilitation training in the treatment of postoperative joint function recovery after development dysplasia of the hip in children. The research results will provide evidence-based medical evidence to support the choice of treatment for the disease.

**Methods:**

Using computer to retrieve PubMed, ScienceDirect, Web of Science, EMBase, Cochrane Library, WANFANG Database, CNKI, and VIP Database, CBM, and using the method of combining mesh words with item words to retrieve the Chinese and English databases, to retrieve the randomized controlled study on the application of traditional Chinese medicine fumigation and washing on the recovery of joint function after development dysplasia of the hip in children. The retrieval time is from January 1990 to January 2021. Two researchers screen and evaluate the quality of the retrieved literatures according to the inclusion and exclusion criteria. In the event of a disagreement, a third researcher will join the discussion to resolve the disagreement. Using Revman 5.3 software to conduct meta-analysis.

**Results:**

This study will compare the application effect and safety of traditional Chinese medicine fumigation and washing as a complementary and alternative therapy and traditional rehabilitation training in the treatment of postoperative joint function recovery after development dysplasia of the hip in children.

**Conclusion:**

The results of this study will be published in an internationally influential academic journal to provide evidence-based medical evidence for the selection of supplement and alternative therapies on the recovery of joint function after development dysplasia of the hip in children.

**Ethics and dissemination:**

This study does not involve specific patients, and all research data comes from publicly available professional literature, so an ethics committee is not required to conduct an ethical review and approval of this study.

**OSF Registration:**

DOI 10.17605/OSF.IO/RUHK5.

## 1 Introduction

Development dysplasia of the hip (DDH), also known as congenital dislocation of the hip, which includes dislocation, semi-dislocation of the hip, and dysplasia of the hip in newborns. It is a common childhood orthopedic disease in clinic.^[1,2]^ The incidence of DDH is influenced by many factors, such as gender, habits, season, fetal position, caesarean, etc, and there are obvious ethnic and regional differences.^[3]^ Relevant data show that the incidence of DDH in China is about 0.9% to 3%, the ratio of men to women is about 1:5 to 1:7. DDH can be one-sided onset, and it can also be on both sides of the disease, one-sided onset of the left side is often more than the right side.^[4]^ At present, the etiology of DDH is not clear, and several theories have been put forward, including mechanical theory, primary dysplasia of the hip, joint capsule and bremsstrahlung relaxation, genetic theory, etc, which are widely recognized.^[5]^ If DDH cannot be treated promptly or correctly, on the 1 hand, it will affect the child's walking gait, leaving a psychological shadow on the child's growth, on the other hand, there is a long-term mismatch between the femoral head and acetabulum, which will cause spinal deformity, osteoarthritis of hip joint, etc, seriously affecting the life quality of the child. Therefore, DDH should be diagnosed and treated early, so as to restore the normal anatomical relationship of hip joint and prevent the occurrence of femoral head necrosis.^[6]^ The treatment principle of DDH is to restore and maintain the concentric position of femoral head and acetabulum as soon as possible. According to different age and severity of the disease, the treatment of children is different. DDH can be diagnosed by ultrasound in early infancy, and the treatment methods include traction or gypsum fixation, with the characteristics of good effectiveness and rapid recovery. The success rate of conservative treatment in children with DDH after toddler stage is reduced, which is partly due to the failure of hip joint to maintain concentric reduction and due to the reduced compliance of children. In the later stage of treatment, femoral head necrosis, reluxation, and other complications often occur. On the other hand, due to the child's age, the degree of hip joint disease is more serious, and simple conservative treatment cannot be effectively reset. Effective reduction requires surgery to remove the soft tissue that hinders the reduction. After the femoral head is reset, the joint capsule needs to be reconstructed to achieve concentric reduction. With the increase of age, the self-remodeling ability of the hip joint in children decreases, and surgical treatment must be used to maintain the concentric position of the femoral head and acetabulum.^[7,8]^ At present, there are many types of surgery to treat the disease, among which pemberton periacetabular osteotomy (PPO) is also known as periacetabular angioplasty, and it often used in the treatment of children with DDH after toddler and achieved better results.^[9,10]^ PPO after surgery needs to be fixed hip gypsum to maintain joint stability, but the fixed time needs to be up to 6 weeks or more, clinically after 6 weeks of gypsum removal clearly found that hip activity is limited. If the joint function is not effectively restored in the early stage, and it can affect the child's early normal daily life after surgery.^[11]^

At present, modern medicine believes that DDH osteotomy surgery can cause hip joint adhesion due to traumatic, insufficient intraoperative soft tissue loosening, delayed functional exercise, long plaster fixation time, etc, which is easy to appear early hip activity is limited, so the treatment method is mainly for the cause of treatment. The treatment methods mainly under anesthesia joint mobilization, continuous passive activity and physiotherapy. The treatment objects are mostly patients with advanced joint stiffness or early joint stiffness treatment failure. For children with limited early activity, functional exercise is often the first choice for rehabilitation.^[12]^ However, due to the young age of the child, and they often need parents to assist in functional exercise. In the process of passive functional exercise, the child's tolerance to pain is lower than that of adults, and ultimately it is more difficult to achieve the desired effect. However, traditional Chinese medicine fumigation and washing has the characteristics of small trauma, small toxic side effects, and simple operation, and it has been reported in the literature that traditional Chinese medicine fumigation and washing has a better effect on improving the restriction of hip activity.^[13]^

Due to the lack of evidence-based medical evidence on the effectiveness and safety of traditional Chinese medicine fumigation and washing as a complementary and alternative therapy on the recovery of joint function after development dysplasia of the hip in children. This study will conduct a systematic review to compare the application effect and safety of traditional Chinese medicine fumigation and washing as a complementary and alternative therapy and traditional rehabilitation training in the treatment of postoperative joint function recovery after development dysplasia of the hip in children and providing evidence-based medical evidence support for finding effective complementary and alternative therapy for the recovery of joint function after development hip dysplasia in children.

## 2 Methods

### 2.1 Protocol register

The protocol of the systematic review has been registered on Open Science Framework (OSF), registration number: DOI 10.17605/OSF.IO/RUHK5 (https://osf.io/ruhk5).

### 2.2 Eligibility criteria

This study does not involve specific patients, and all research data comes from publicly available professional literature, so an ethics committee is not required to conduct an ethical review and approval of this study.

#### 2.2.1 Type of study

The randomized controlled study of traditional Chinese medicine fumigation and washing as a complementary and alternative therapy on the recovery of joint function after development dysplasia of the hip in children. The language of the study is set to Chinese or English.

#### 2.2.2 Population

Children who are clinically diagnosed as DDH and treated with PPO (all children had indications for open reduction and acetabular osteotomy). The diagnostic criteria refer to the diagnostic criteria for DDH in the “*Guidelines for the Diagnosis and Treatment of Development Dysplasia in the Hip (2009 Edition)*” formulated by the Orthopedic Branch of the Chinese Medical Association. The gender, age, and course of the disease are not limited.

#### 2.2.3 Inclusion criteria

(1) Meet the diagnostic criteria of DDH.
(2) The children are older than 3 years old and younger than 7 years old.
(3) Children who underwent unilateral Pemberton acetabular osteotomy for the first time.
(4) There are no other diseases in the bilateral hip joints.
(5) The muscle strength around the hip joint is normal, and there is no neuropathic joint disease.
(6) The relevant examination results of the child meet the surgical indications for open reduction and acetabular osteotomy, and there are no surgical contraindications.
(7) Meet relevant ethical requirements.

#### 2.2.4 Exclusion criteria

(1) It did not meet the diagnostic criteria of DDH.
(2) There are diseases of blood system, cardiovascular and cerebrovascular system, nervous system, and so on.
(3) There are diseases that affect the development of hip joint, such as poliomyelitis sequelae, congenital equinovarus, and so on.
(4) There is traumatic dislocation of the hip.
(5) There are secondary pathological dislocation of the hip, such as rickets, suppurative arthritis of the hip, and so on.
(6) Incomplete data, loss of follow-up, or uncooperative follow-up.
(7) Review, case study, animal experiment, and other nonrandomized controlled studies.

#### 2.2.5 Types of interventions

Interventions for the control group: The children receive routine examination and nursing before treatment, and routine functional exercises after the plaster is removed after the operation.

Interventions for the experimental group: The children receive routine examination and nursing before treatment. After the plaster is removed after the operation, they were treated with traditional Chinese medicine fumigation and washing combined with routine functional exercises, or the traditional Chinese medicine fumigation and washing is used alone. There are no restrictions on the selection, dosage, frequency of treatment, and overall treatment time for traditional Chinese medicine fumigation and washing.

#### 2.2.6 Outcome indicators

The primary outcome indicators include:

(1) Makay hip function score: follow-up the patients, observation of patients after surgery ≥12 months of hip function score, a total score of 100 points, excellent: Makay score ≥ 90 points, joint stability, no pain, lameness, Trenderlenburg negative; good: score 80 to 89 points, joint stability, no pain, slight lameness, slightly restricted movement, Trenderlenburg negative; basic recovery: score 70 to 79 points, joint stability, no obvious pain, limp, Trenderlenburg positive; poor: score < 70, pain significant, trenderlenburg positive.
(2) Acetabular index: on the pelvic X-ray images before operation and at the last follow-up, the line connecting the apex of bilateral triangular cartilage is H-line, and the angle between the line connecting the external superior edge of triangular cartilage and the external superior edge of acetabulum and H-line is acetabular index.

The secondary outcome indicators include:

(1) The time of surgery and the amount of bleeding during surgery.
(2) Safety indicators: adverse reactions in the process of traditional Chinese medicine fumigation and washing after surgery, such as: skin allergies, postoperative complications, such as stoic nerve damage. Re-dislocation of the hip joint occurs.

### 2.3 Data sources and search strategies

Mainly by means of computer retrieval, in CBM, VIP, CNKI, WANFANG Database, PubMed, ScienceDirect, Web of Science, EMBase, Cochrane Library. A randomized controlled study on the application of traditional Chinese medicine fumigation and washing to the recovery of joint function after development dysplasia of the hip in children is collected comprehensively. The retrieval time is from January 1990 to January 2021. Using the methods of combing mesh words and item words, and through logical “OR” and “AND,” the search words are combined to retrieve the literature. The scope of the retrieve is the title and summary. Manually search all the detected reviews, meta-analysis and references of included research literature to improve the recall rate of the literature. The results of literature retrieval in PubMed database by subject words and free words are shown in Table 1.

**Table 1:** Results of literature retrieval in PubMed database by subject words and free words.

Number	Search items
#1	Search: (Medicine, Chinese traditional[MeSH Terms]) OR (Traditional Chinese medicine[Title/Abstract]) OR (Chinese herbal medicine [Title/Abstract]) OR (Chung I Hsueh[Title/Abstract]) OR (Hsueh, Chung I[Title/Abstract]) OR (Traditional Medicine, Chinese[Title/Abstract]) OR (Zhong Yi Xue[Title/Abstract]) OR (Chinese traditional medicine[Title/Abstract]) OR (Chinese Medicine, traditional[Title/Abstract]) OR (Traditional tongue diagnosis[Title/Abstract]) OR (Tongue diagnoses, traditional[Title/Abstract]) OR (Tongue diagnosis, traditional[Title/Abstract]) OR (Traditional tongue diagnoses[Title/Abstract]) OR (Traditional tongue assessment[Title/Abstract]) OR (Tongue assessment, traditional[Title/Abstract]) OR (Traditional tongue assessments[Title/Abstract])
#2	Search: ((Fumigation and Washing) [Title/Abstract])
#3	Search: (Complementary therapies[MeSH Terms]) OR (Therapies, complementary[Title/Abstract]) OR (Therapy, complementary[Title/Abstract]) OR (Complementary medicine[Title/Abstract]) OR (Medicine, complementary[Title/Abstract]) OR (Alternative medicine[Title/Abstract]) OR (Medicine, alternative[Title/Abstract]) OR (Alternative therapies[Title/Abstract]) OR (Therapies, alternative[Title/Abstract]) OR (Therapy, alternative[Title/Abstract])
#4	Search: (Development dysplasia of the hip[Title/Abstract]) OR (Congenital dislocation of the hip[Title/Abstract])
#5	Search: (Joint function[Title/Abstract]) OR (Joint functions[Title/Abstract])
#6	Search: (Child[Title/Abstract]) OR (Children[Title/Abstract])
#7	#1 AND #2 AND #3
#8	#4 AND #5 AND #6
#9	#7 AND #8

### 2.4 Data collection and analysis

**Figure 1:**
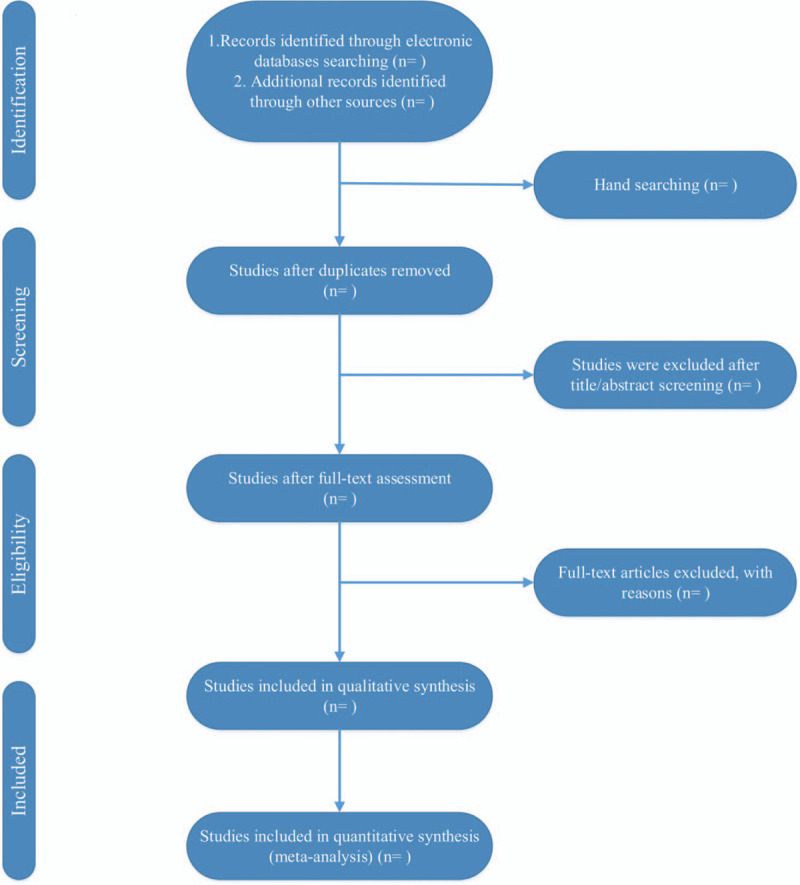
The documents retrieval process for Chinese and foreign language databases.

The literature is independently screened by 2 researchers. By reading the topics and abstracts and further reading the full text, excluding literature that do not meet the inclusion and exclusion criteria, extracting and cross-checking the data using a unified Excel extraction form for the literature entering meta-analysis, which can be resolved with the assistance of the third researcher if a disagreement is encountered. The contents of the data extraction mainly include: study source (topic, author, year of publication, magazine, etc), study design (whether the specific method of random allocation is described, whether the allocation is hidden, whether the blind method is used, whether the information about patients’ loss of follow-up or withdrawal is described), study subjects (age, sex ratio, number of cases, interventions, drug dose, course of treatment, etc), outcome indicators (main outcome indicators and secondary outcome indicators). The documents retrieval process for Chinese and foreign language databases is shown in Figure 1.

### 2.5 Quality assessment

Two researchers carefully read the relevant sections of the Cochrane Intervention System Evaluation Manual to conduct bias risk assessment of inclusion study based on the manual's bias risk assessment tool for randomized controlled studies. Each literature is evaluated in 7 ways:

(1) Whether there is a description of the random sequence generation method.
(2) Whether there is a description of allocation hidden method, and whether the allocation hidden is correct.
(3) Whether implement blind methods on researchers and subjects.
(4) Whether implement blind methods on the outcome evaluator.
(5) Whether complete results data are reported.
(6) Whether the study results are reported selectively.
(7) Whether there are factors that give rise to other bias risks.

Based on the above, “yes,” “unclear,” and “no” are used as the evaluation results. The study is considered to have a low bias risk, if the evaluation results of all entries are low bias; medium bias risk is considered, if the evaluation results of one or more entries are unclear; and high bias risk is considered, if one or more entries are high bias.

### 2.6 Statistical method

Using RevMan 5.3 software to perform statistics and analysis of relevant data.

#### 2.6.1 Data synthesis

The extracted data is statistically analyzed using Revman 5.3 software. The continuous variable data included in the study are based on mean difference as an effectiveness analysis statistic and calculates its 95% confidence interval, setting *P* < .05 is statistically significant. If the included study provides only mean and standard deviation (mean ± SD) of each clinical indicator before and after treatment, statistical analysis is required to estimate the change effect in each clinical indicator using the relevant tools of Cochrane Handbook.

#### 2.6.2 Heterogeneity test

Due to a variety of factors, such as the difference of study design, study objects, interventions, clinical outcome indicators, statistical analysis methods, etc, there will be more or less differences between studies, this difference is called heterogeneity, clinical heterogeneity, methodological heterogeneity, and statistical heterogeneity. The studies normally included should be homogeneity and the aggregated results should be credible and reliable. Therefore, before meta-analysis, the clinical results included in the study need to be tested for heterogeneity, and the appropriate effect model should be selected based on the test results to analyze the possible causes of heterogeneity and determine whether the meta-analysis is feasible. In this study, the heterogeneity test is carried out on the clinical indicators included in the study using the chi-square test (the test standard is set to α = 0.5), and the heterogeneity is quantitatively judged by combining the amount of *I*^2^ values. If the chi-square test results show *P* ≥ .1, *I*^2^ ≤ 50%, suggesting that there is no heterogeneity or less heterogeneity between studies, the fixed effect model is used for analysis, on the other hand, when the card test results show *P* ≤ .1, *I*^2^ ≥ 50%, suggesting that the heterogeneity between studies is larger, selectable random effect model analysis. For heterogeneity treatment, in addition to selecting random effect model, the source of heterogeneity can be understood by subgroup analysis and sensitivity analysis, etc to understand the source of heterogeneity. If none of the above can explain the reasons for heterogeneity, the meta-analysis can only be descriptive or discarded.

### 2.7 Sensitivity analysis

Sensitivity analysis is primarily used to determine whether the decision-making of each step is robust, whether it has an impact on the combined effect, or to analyze the sources of heterogeneity between studies.^[14]^ In the process of meta-analysis, there are many decision nodes that may require sensitivity analysis, including:

(1) Changing study retrieval strategies;
(2) Formulating the different inclusion study criteria;
(3) Including the different study objects;
(4) Choosing different interventions measures or control groups;
(5) Changing the clinical outcome indicators of the included studies;
(6) Adopting different statistical analysis methods.

When analyzing the effects of a single study on the amount of combined effect, a single study can be excluded in turn to see whether the amount of effect after the elimination of the study has changed that compared with the previous. If the value of the outcome indicator is significantly different, it is suggested that the study has a greater impact on the total effect, and the full text should be read carefully to analyze the reasons. When analyzing the effects of a single study on heterogeneity, if the heterogeneity is significantly reduced after excluding a study, it is considered that the study is the main cause of heterogeneity, and the study should be carefully read and analyzed again, reconsidered whether to include it, and explained in the discussion.

### 2.8 Subgroup analysis

For the heterogeneous outcome indicators, we will use the method of subgroup analysis to solve this problem. The types of drugs used in traditional Chinese medicine fumigation and washing, the length of fumigation and washing, the age of the patient, dysplasia of the hip is unilateral or bilateral, the gender of the patient.

### 2.9 Publication bias

Publication bias mainly refers to the fact that the study results with statistical significance in the meta-analysis are easier to publish or report than those without significant or invalid results. Funnel plots are often used in RevMan 5.3 software to analyze that whether there is publication bias. Funnel plots refer to a simple scatter figure drawn with the effect amount of a single study as the horizontal coordinate and the standard error of the effect number pair or the standard error of the effect quantity as the ordinate. The more dispersed the scattered distribution, the smaller the sample size, and the more concentrated the scatter distribution, the larger the sample size. If the figure is symmetrical, there is no bias, on the contrary, if the figure is asymmetric, there is bias. This study analyzes publication bias by examining the asymmetry of the funnel plot.

### 2.10 Grading the quality of evidence

Based on the results of the meta-analysis, the evaluation of important outcome indicators was carried out using the classification method system of evidence quality and recommendation strength launched by the GRADE working group in 2004 (hereinafter referred to as GRADE system). Use GRADE pro 3.6.1 software for analysis. Among them, RCT was initially rated as high-quality evidence, and its quality can be reduced due to 5 factors (risk of bias, inconsistency, indirectness, accuracy, and other factors), and finally rated as high, medium, low, and very low grade. At the same time, combining the pros and cons of intervention measures, limitations, suitable population, cost and health care, and other factors, the recommendations are finally formed into 2 levels of strong and weak.

## 3 Discussion

DDH is one of the common hip joint diseases in children, and the prevalence rate in China is about 3.8%. Twenty percent of the patients had a family history of DDH, with a higher proportion of female patients.^[15]^ DDH children who do not receive timely professional treatment, and the disease gradually worsens with the increase of age. The clinical manifestations have limb shortening, limb instability, lameness, etc, which is one of the main diseases leading to physical disability in children.^[16]^ At present, surgery is the main means of treating older DDH, especially for patients with severe lesions of the hip joint and surrounding soft tissues. Pemberton periacetabular osteotomy is one of the commonly used surgical methods at present. This osteotomy method maintains the relative stability of the pelvis through incomplete osteotomy, which mainly improves the acetabulum's tolerance of the femoral head, increase the coverage of the femoral head, and it can effectively restore concentric reduction.^[17]^

After the surgery of DDH, it needs to be fixed with external fixation method to maintain the concentric position of the hip joint for up to 6 to 8 weeks. During this process, the joint capsule and the connective tissue around the joint change from a loose combination to a dense combination, and the tissue shrinks and adheres. Thickness limits the relaxation of the muscles around the hip joint for a long time, and the muscle loses its elasticity, resulting in a decrease in the maximum length of muscle relaxation. In order to ensure functional exercise, the limitation of hip joint movement often occurs.^[18]^ To solve the problem of limited joint activity after DDH is the premise and focus of hip joint rehabilitation. Modern medicine has not many treatment methods for limited joint activity after DDH, to improve the joint activity routine, routine functional exercise, manual mobilization under anesthesia, and continuous passive activity has certain effects. Manual mobilization under anesthesia needs to bear the risk of anesthesia again, and the cost is high. Early patients’ family members are less willing to treat, and they prefer to choose functional exercise. Most patients with limited joint activity in the early stage do not get effective treatment and have late joint stiffness symptoms, so the hospital surgery was relieved. The main reason why the early joint activity restriction is not improved in time is mainly due to the poor fit of children, so that the effect of simple functional exercise is not significant, and it cannot achieve better-expected effect. The continuous passive activity to adjust the angle and strength to prevent re-dislocation, so when the plaster is removed for external fixation after operation, this treatment method cannot be selected. Traditional Chinese medicine fumigation and washing belongs to the category of Chinese medicine external treatment, which can directly act on the focus. It has the functions of relaxing muscles and tendons and removing obstruction from meridians, activating blood to eliminate stagnation. It has achieved good effects in relieving joint disorders. Modern pharmacology has also confirmed that many of the Chinese herbal medicine can relax muscles and tendons and removing obstruction from meridians, activating blood to eliminate stagnation, which is conducive to tissue metabolism, and effectively relieve the tension of deep muscles, and thus play a role in relieving spasms and pain.^[19,20]^ However, there is a lack of high-quality evidence support, so the clinical application effect of traditional Chinese medicine fumigation and washing is controversial. This study will be the same type of randomized control study that meets the inclusion criteria. This study will conduct a systematic review to compare the application effect and safety of traditional Chinese medicine fumigation and washing as a complementary and alternative therapy and traditional rehabilitation training in the treatment of postoperative joint function recovery after development dysplasia of the hip in children. The results of this study will be helpful for the selection of treatment methods on the recovery of joint function after developmental dysplasia of the hip in children.

## Author contributions

**Conceptualization:** Jianping Lin.

**Data curation:** Weipeng Zeng, Hanru Mao, Gang Zhou, Kaiqiu Wu, Xinping Liao.

**Formal analysis:** Weipeng Zeng, Hanru Mao, Gang Zhou, Linqian Yun.

**Funding acquisition:** Jianping Lin.

**Methodology:** Weipeng Zeng, Hanru Mao, Gang Zhou.

**Resources:** Weipeng Zeng, Gang Zhou, Kaiqiu Wu, Linqian Yun.

**Software:** Hanru Mao, Gang Zhou, Xinping Liao.

**Writing – original draft:** Weipeng Zeng, Hanru Mao, Gang Zhou, Kaiqiu Wu, Xinping Liao, Linqian Yun.

**Writing – review & editing:** Jianping Lin.
